# The impact of RHDV-K5 on rabbit populations in Australia: an evaluation of citizen science surveys to monitor rabbit abundance

**DOI:** 10.1038/s41598-019-51847-w

**Published:** 2019-10-23

**Authors:** Tarnya E. Cox, David S. L. Ramsey, Emma Sawyers, Susan Campbell, John Matthews, Peter Elsworth

**Affiliations:** 1 Vertebrate Pest Research Unit, New South Wales Department of Primary Industries, 1447 Forest Road Orange, New South Wales, 2800 Australia; 2 Arthur Rylah Institute for Environmental Research, Department of Environment, Land, Water and Planning, PO Box 137, Heidelberg Victoria, 3084 Australia; 3 Centre for Invasive Species Solutions, Building 22, University of Canberra, University Drive South, Bruce, Australian Capital Territory 2617 Australia; 4 Biosecurity and Sustainability, Department of Primary Industries and Regional Development, 444 Albany Hwy, Albany, Western Australia 6330 Australia; 5 Department of Economic Development, Jobs, Transport and Resources, 147 Bahgallah Road, Casterton, Victoria 3311 Australia; 6 Pest Animal Research Centre, Department of Agriculture and Fisheries, PO Box 102, Toowoomba, Queensland 4350 Australia; 7 School of Biological Sciences, Molecular Life Sciences Building, University of Adelaide, North Terrace, Adelaide, 5005 Australia

**Keywords:** Environmental sciences, Environmental social sciences

## Abstract

The increasing popularity of citizen science in ecological research has created opportunities for data collection from large teams of observers that are widely dispersed. We established a citizen science program to complement the release of a new variant of the rabbit biological control agent, rabbit haemorrhagic disease virus (RHDV), known colloquially as K5, across Australia. We evaluated the impact of K5 on the national rabbit population and compared citizen science and professionally-collected spotlight count data. Of the citizen science sites (n = 219), 93% indicated a decrease in rabbit abundance following the release of K5. The overall finite monthly growth rate in rabbit abundance was estimated as 0.66 (95%CI, 0.26, 1.03), averaging a monthly reduction of 34% at the citizen science sites one month after the release. No such declines were observed at the professionally monitored sites (n = 22). The citizen science data submissions may have been unconsciously biased or the number of professional sites may have been insufficient to detect a change. Citizen science participation also declined by 56% over the post-release period. Future programs should ensure the use of blinded trials to check for unconscious bias and consider how incentives and/or the good will of the participants can be maintained throughout the program.

## Introduction

Citizen science, the participation of members of the public in scientific endeavors, has become increasingly prominent in contributing to ecological research. The ability to engage a potentially large team of widely-dispersed observers has created opportunities for data collection over spatial and temporal scales that are difficult to replicate with conventional monitoring programs^1^. The rise of citizen science programs has also been greatly enabled by the development of internet-based data-entry technologies that has allowed citizen scientists to submit location-based data electronically. In addition to enabling broad scale data collection, citizen science programs also have the added benefit of increasing positive engagement by the public with scientific research^2^. Citizen science data are now routinely used by researchers to model the distributions, abundance and species richness of plants and animals^2–4^.

Citizen science has resulted in unprecedented access to data to inform ecological research, but there can be inherent limitations with citizen-science-sourced data. Participants in citizen science programs often have no training in the scientific method and may have limited expertise in the subject matter, which potentially results in the submission of data that are inaccurate or of poor quality. The ability to make anonymous contributions to data repositories can also result in misleading or malicious data being submitted^5^. Lack of commitment by participants may also result in gaps in the data. These and other issues have been used to suggest that citizen science sourced data should not be considered for serious scientific research^5^. Hence, many studies have been devoted to the evaluation of citizen science data, usually by comparison with similar data collected by standard scientific methods^2,3^.

Rabbits are a significant agricultural and environmental pest in both Australia and New Zealand. In Australia they threaten 321 species of plant and animal, 75 ecological communities and contribute to production losses of >AUD$209 million annually, despite ongoing rabbit control. Successful landscape-scale management of rabbits has only been achieved through the use of the self-disseminating biological control agent’s myxoma virus (MYXV, Family Poxviridae) and rabbit haemorraghic disease virus (RHDV, Family Caliciviridae). The last release of a biological control agent (RHDV) was in 1995 and over time, virus efficacy has waned due to acquired and genetic immunity and the interference of an endemic benign strains of calicivirus.

A new Korean variant of RHDV, Incheon 08Q712 (commonly and hereafter referred to as K5), was approved and registered for use as a rabbit biocontrol agent in April 2016. K5 was selected for release as it can overcome the protective effects of a benign calicivirus, Rabbit Calicivirus Australia-1 (RCV-A1)^6^, which confers partial temporal protection to infection by early Australian variants of RHDV^7^. RCV-A1 is predominantly found in the cool, high rainfall (573 ± 217 mm mean annual rainfall) areas of Australia^8^. RCV-A1 hampered the impact and effectiveness of the first RHDV release in Australia in 1996; with greater rabbit population reductions occurring in hot, dry areas compared to cool, wet areas^9,10^.

To maximise the potential impact of this new strain on rabbit populations, it was decided to release the K5 virus at many locations, distributed widely over the known range of rabbits in Australia in March 2017. To help achieve this, a citizen science release program was established. The program was designed to encourage participation in the release and the use of best practice follow-up control, and to utilise the growing interest in citizen science to capture data on the impact of K5 on rabbit populations across the country. Using citizen scientists to assist with the release would help ensure that the virus could be released widely, and near-simultaneously, in many locations across Australia. This would be logistically and financially impractical to achieve using the network of professional ecologists in state agencies involved in the K5 release program.

Here we describe how we used citizen science to collect data in a national pest management program. We analyse the immediate impact of the release of RHDV-K5 using the citizen science collected data (pre- and post-release spotlight counts) and we compare the citizen science data with the professional data by using the professional data as a baseline. We discuss the benefits and pitfalls of the use of citizen scientists in these types of programs and make recommendations on how citizen science could be better utilized.

## Methods

In order to maximise the distribution of K5 we established a citizen science program to supplement the national monitoring program. The national program consisted of 22 intensively monitored sites (11 pairs of release and control sites) (Supplementary Information Fig. S3). Statistical modelling by^11^ suggested that these 11 paired sites were sufficient to detect a change in rabbit population due to K5. However, given the highly variable and disconnected nature of Australia’s rabbit population, we felt it warranted to establish as many release and monitoring sites as possible to adequately measure the variable impact of K5 across a range of “susceptibility landscapes” (areas with varying levels of benign calicivirus presence, genetic resistance and acquired immunity of rabbits to RHDV and rainfall patterns).

### Encouraging citizen science participation

To stimulate interest in the upcoming release program, we ran a series of information sessions. Information sessions were held throughout the country from August 2015-November 2016 and consisted of a presentation on the project (expected outcomes and how people could get involved), followed by a presentation on best-practice rabbit management and follow-up control techniques (e.g. warren ripping, warren fumigation, poisoning). Media releases and a television segment on the Australian Broadcasting Commission’s national Landline program also exposed the program to a wider audience than the roadshows alone could reach.

### Expressions of interest process

Land managers were asked to submit an online Expression of Interest (EOI) if they wished to participate in the program. All participants were requested to undertake a three night spotlight count of their rabbit population one month before the K5 release. Participants were asked to collect dead rabbits (where possible) after the release of K5 and to redo their rabbit population assessment in the same manner four weeks after they released (approximately early April 2017). The EOI form was open for submission from December 2015-May 2016 and was available on the PestSmart website (www.pestsmart.org.au).

### Citizen science site selection

**Figure 1:**
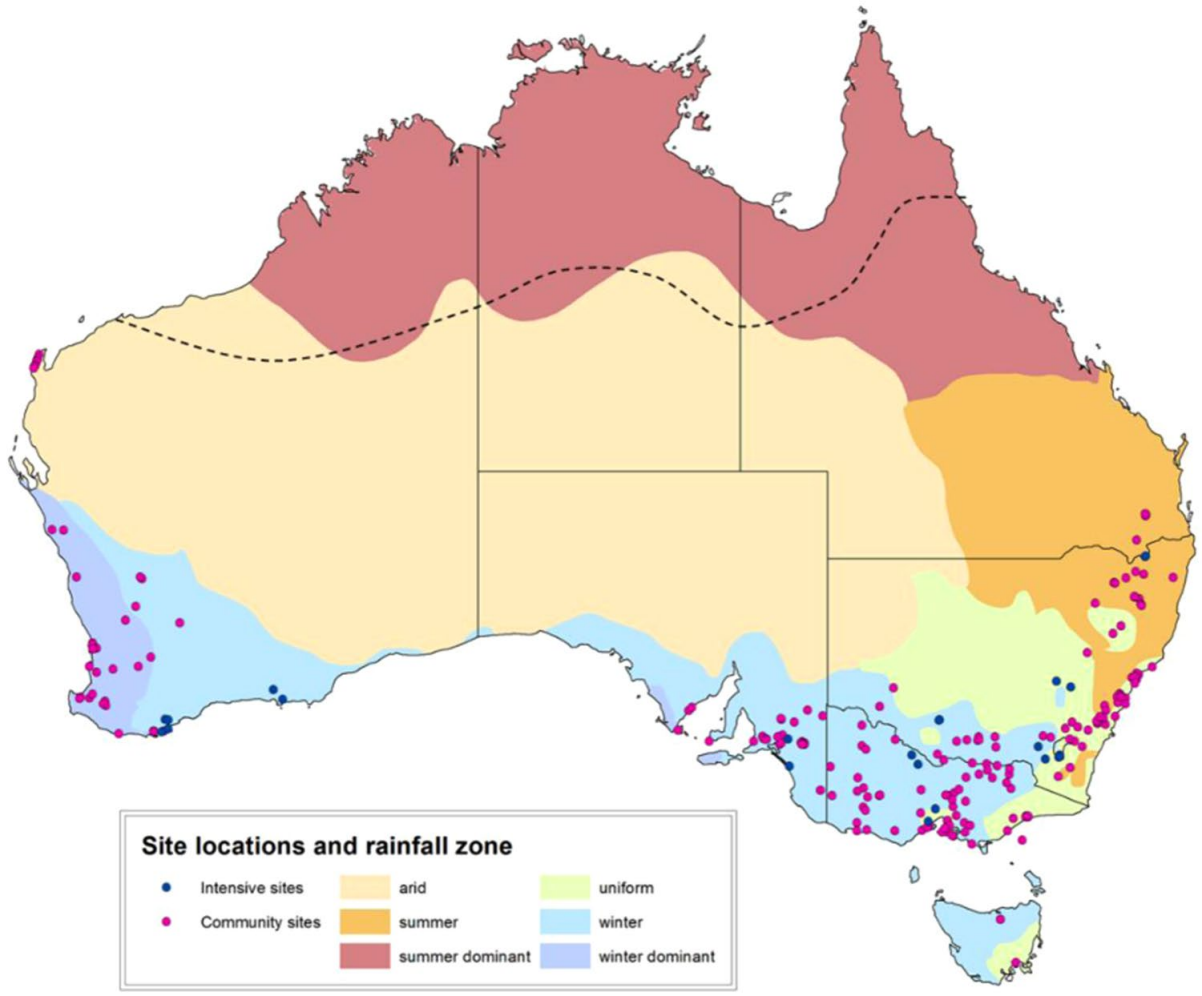
Locations of citizen science release sites (pink circles) and professionally monitored sites (blue circles) overlaid on the major seasonal rainfall zones. Dashed line indicates northern limit of rabbit distribution. Map created in ArcGIS Desktop, Release 10.3.1.

Each state and territory in Australia had a lead coordinator (State Lead) who undertook site selection for their region and acted as a point of contact for local enquiries. Sites that were within 50 km of an intensive monitoring site were excluded, except where that intensive site was designated as a release site. State Leads were provided with a list of national criteria on which to base their initial site selection (see Supplementary Information). The locations of each site were matched to a major seasonal rainfall zone based on a classification of 100 years of seasonal rainfall data from 1900–1999^12^ with each zone describing the season with predominant rainfall and the arid zone describing the region with generally low rainfall (<250 mm). No sites were based in areas of summer dominant rainfall and hence, only 5 classifications were used. The locations of the 219 citizen science release locations used in the analysis are given in Fig. 1.

### Data collection

To facilitate and streamline data entry we modified an existing resource, RabbitScan, to allow participants to upload their data electronically. RabbitScan was available both on the web and as a smart device (smartphone or tablet) app. Participants could upload their spotlight counts or report dead rabbits and request a sampling kit. Data from a site was considered complete if at least two nights pre- and post-release spotlight data were completed and a spotlight transect length of at least 100 m was used.

### Site support and communication

Participants were provided with a number of resources to assist them with virus distribution and data collection, including; a fridge magnet with their site number, a K5 release booklet (containing information on rabbits in Australia, the history of rabbit biocontrol and background on the RHD-boost project, five pre-labelled sample collection tubes with a reply-paid envelope, five pairs of gloves, a RabbitScan card to record their login details, a sample collection procedure which also included information on human health and zoonotic diseases and a USB of how-to videos (information on how to do a spotlight count, how to use RabbitScan to submit data and record disease, how to collect samples from a rabbit and how to conduct follow-up controls such as baiting with poison, fumigation and warren ripping using best-practice techniques).

### Professional monitoring data

Monitoring of rabbit abundance by professional ecologists was undertaken at 22 sites located across Australia (Fig. 1). Sites within each state were organized as pairs with one site serving as the K5 release site and the other as a non-release (control) site (i.e. 11 pairs in total). Each pair of sites was spatially matched and while every effort was made to ensure sites were separated by a minimum distance of 25 km, this was not achieved at two of the paired sites. All sites were subject to a three night spotlight count four times per year (middle of each season). Professional monitoring data used for this analysis were collected in January/February (pre-release) and March/April (post-release) to match the citizen science data collection period.

### Analysis

The rabbit counts at each site *i* and survey period *t* (i.e. pre- or post-release) and occasion *k* (*y*_*itk*_) were corrected for imperfect detectability to estimate absolute rabbit abundance at site *i* and survey period *t* (*N*_*it*_) using a binomial *N*-mixture model^13^. The *N*-mixture model uses the information on the *k* repeated counts during a survey to estimate detection probability for survey period *t*. The *N*-mixture model for rabbit abundance was given by

1 $$\begin{array}{lll}{y}_{itk} &  \sim  & Bin({p}_{it},{N}_{it})\\ {N}_{it} &  \sim  & Poisson({\mu }_{it})\\ {\rm{logit}}({p}_{it}) &  \sim  & N({\alpha }_{p},{\sigma }_{p})\end{array}$$

where *y*_*itk*_ are the counts of rabbits at site *i* during survey period *t* and occasion *k*, *p*_*it*_ is the detection probability of rabbits at site *i* and survey period *t* and *μ*_*it*_ is the expected population abundance of rabbits. For this model, the (logit) detection probability at each site *i* and survey period *t* was assumed to be normally distributed with mean *α*_*p*_ and standard deviation *α*_*p*_. For the pre-release survey period (*t* = 1), the expected rabbit abundance at site *i* was a function of the length of the transect monitored at each site (*T*_*i*_), which was included as an offset to account for the variable length of transects monitored at each site.

2 $$\begin{array}{c}\log ({\mu }_{i1})={\beta }_{i}+\log ({T}_{i})\end{array}$$

Hence, the parameters *β*_*i*_ are the estimate of rabbit abundance per spotlight km for the pre-release survey period, for each site *i*. The estimate of rabbit abundance for the post-release period (*t* = 2) was similar to that for the pre-release period with the addition of a term for the rate of increase *r*_*i*_ of the rabbit population between the pre- and post-release periods, for each site *i*. However, the difference in the time elapsed between the pre-release and post-release surveys varied markedly among citizen science monitored sites from 1 to 160 days, while approximately 90 days had elapsed between pre- and post-release surveys for the professionally monitored sites. This was accounted for by multiplying the growth rate *r*_*i*_ by the elapsed time (in months) between pre- and post-release surveys (*δ*_*i*_) (Eq. 3). Hence, Eq. (3) represents a simple exponential growth trend model where *r*_*i*_ was equivalent to the monthly growth rate and exp(*r*_*i*_) the finite (proportional) monthly growth rate (Humbert *et al*. 2009).

3 $$\begin{array}{rcl}\log ({\mu }_{i2}) & = & {\beta }_{i}+{r}_{i}{\delta }_{i}+\,\log ({T}_{i})\\  &  & {\beta }_{i} \sim N({\gamma }_{z},{\sigma }_{z})\\  &  & {r}_{i} \sim N({\eta }_{z},{\sigma }_{r})\end{array}$$

For the citizen science monitored sites, we structured the estimates of rabbit abundance *β*_*i*_ and monthly growth rates *r*_*i*_ for each site so that they were a function of rainfall zone by using a hierarchical random effects linear model. Here, *γ*_*z*_ and *η*_*z*_ are the (log) mean abundance of rabbits and monthly growth rates within each rainfall zone *z* with *σ*_*z*_ and *σ*_*r*_ the respective standard deviations.

For the professional monitoring data, there were insufficient sites to estimate rabbit abundance within each rainfall zone. Hence, rabbit abundances were estimated for each site and survey period separately, using a non-hierarchical model. Sites were classified on whether they were a K5 release site or a non-release (control) site. As for citizen science monitored data, the length of the spotlight transect (*T*_*i*_) was included as an offset in the abundance part of the *N*-mixture model.

### Comparisons of citizen science and professional monitoring data

One major drawback with obtaining inferences about the effect of the K5 release from the citizen science monitoring data was that there were no non-release (blinded control) sites for comparison. Hence, changes in rabbit abundances due to the release of K5 were confounded with survey time (pre and post-release periods). We attempted to resolve this by comparing the results obtained from the citizen science data with results from similar data obtained from the professionally monitored sites that included paired K5 release and non-release sites.

We evaluated whether the estimates of the impact of the K5 release derived from the citizen science monitored data were consistent with that obtained from the professionally monitored data using a Bayesian hierarchical modelling approach. This approach attempts to assess the consistency of the citizen science monitored data by comparing it with a reliable baseline dataset (here the professionally monitored data). Similar approaches at integrating citizen science data and professional data to assess the reliability of citizen science data have been dubbed “Bayesian data reconciliation”^3,14^. We attempted to estimate the discrepancy in the estimates of the monthly growth rate *r*_*i*_ from the citizen science data by modelling the citizen science *r*_*i*_ estimates as a function of the average monthly growth rate estimated from the professional data

4 $$\begin{array}{rcl}{r}_{i}^{p} & \sim  & N({\mu }_{rp},{\sigma }_{rp})\\ {r}_{i}^{c} & \sim  & N(\kappa +{\mu }_{rp},{\sigma }_{rc})\end{array}$$

where ${r}_{i}^{p}$ and ${r}_{i}^{c}$ are the estimates of the monthly growth rates for each site between pre- and post-release periods from the professional and citizen science data, respectively, with *μ*_*rp*_ the overall mean monthly growth rate of the professional data and *σ*_*rp*_ and *σ*_*rc*_ the standard deviations in the growth rate for professional and citizen science data, respectively. The parameter *κ* represents the estimate of the discrepancy of the citizen science *r*_*i*_ with the mean of the professional *r*_*i*_. In addition, exp(*κ*) can be interpreted as the ratio of the finite monthly growth rates of the citizen science and professionally monitored data. Furthermore, by having separate standard deviation terms, the discrepancy in the variances of the growth rates between citizen science and professional data can also be compared. Similar to the growth rate, estimates of the detection probability of rabbits were compared using a similar model to (4) above applied to the *p*_*it*_.

### Model fitting

Models were fitted separately to the citizen science and professionally monitored data using the Bayesian Markov Chain Monte Carlo (MCMC) software *Stan*^15^ Mildly informative *N*(0, 5) priors were used for the parameter estimates for the (log) rabbit abundances (i.e. *γ*_*z*,*t*_) with half-*t*_4_ priors used for the standard deviation parameters. In addition, an informative prior for the mean *logit* detection probability parameter was also used, specified as *N*(0, 1). This assumes that the overall average detection probability was unlikely to be either very high or very low with 95% of the mass occurring between 0.12 and 0.88. This prior was based on previous studies on spotlight counts for detecting rabbits that estimated the detection probability to be 0.5–0.7^16,17^. The convergence of the MCMC algorithm was assessed using the scale-reduction diagnostic of Brooks & Gelman^18^, and by visual inspection of parameter traceplots. A burn-in of 2000 iterations was undertaken, followed by sampling from three independent Markov chains with different starting values for 1000 further iterations. Hence, a total of 3000 samples from the three chains were retained for inference.

### Approvals

On submission of an EOI and on signing up to the Rabbit Scan portal, a participant agreed to have their data used by the program and to be contacted by staff at any stage in relation to their participation and /or data submission. All participants were over 18 years of age. As rabbits are a pest animal and listed as a Key Threatening Process in Australia^19^, landholders are required by legislation in all States and Territories to manage rabbits to low numbers. Participation in a state government-run pest animal program does not require human ethics approvals. Pest rabbit management, including monitoring of populations, falls under “standard on-farm pest management” practices for participants. Procedures for rabbit management by landholders are governed by Standard Operating Procedures and Model Codes of Practice (http://pestsmart.org.au/tag/rabbit-sop/) and the Australian Pest Animal Strategy^20^. All virus release was undertaken in accordance with the relevant State or Territory rules and procedures around the use of RHDV. We conducted our research under Orange Animal Ethics research authority approval #ORA 14/17/01.

## Results

### Citizen science participation

We received 756 expressions of interest, many of which encompassed multiple groups, resulting in 1066 potential release sites and many more participants (see Supplementary Information Fig. S1). After the site selection process the number was reduced to 738 citizen science release sites nationally (see Supplementary Information Fig. S2).

### Citizen science monitoring data

The community submitted 420 pre-release records and 236 post-release records through RabbitScan although only 131 of these were complete pre- and post-release records for specific sites. An additional 87 complete records were obtained by directly contacting 206 participants via email or telephone. Of the 738 selected sites, 155 EOIs confirmed that they did not release RHDV1 K5. Of the remaining sites only 323 citizen science sites were confirmed to have released RHDV1 K5. The remaining sites (n=260) did not provide any information on their release status despite repeated phone calls and email correspondence. Of the 323 confirmed release locations only 219 provided complete pre- and post-release monitoring data.

### Citizen science data

Fitting the models separately to the citizen-science- and professionally-monitored sites indicated that the estimates of initial rabbit abundance on citizen science sites were not that dissimilar to those on the professional sites, although citizen science sites tended to have a higher proportion of sites with low initial rabbit abundances (<2.7 rabbits/km) than professional sites (Table 1). However, citizen science sites were generally conducted on much shorter transects than professional sites (median of 1 km vs 6 km) and post-release surveys were generally conducted less than two months following the pre-release surveys, compared with around three months for professional monitoring sites (Table 1).

**Table 1:** Summary statistics (Minimum – min; 1^st^ quartile – 1^st^ Qu; Median, Mean, 3^rd^ quartile – 3^rd^ Qu; Maximum – max) for the variables initial (log) rabbit abundance (rabbits/km), transect length and elapsed time between pre- and post-release surveys for the citizen science and professional monitored sites.

Variable	Site	Min.	1st Qu.	Median	Mean	3rd Qu.	Max.
Initial abundance (rabbits/km)	Citizen science	−1.7	1.7	2.9	2.8	4.1	6.9
	Professional	1.0	1.4	2.5	2.7	3.4	6.0
Transect length (km)	Citizen science	0.1	1.0	1.0	2.5	2.5	44.0
	Professional	2.0	2.4	6.2	7.7	11.2	17.8
Elapsed (months)	Citizen science	0.0	1.4	1.8	1.8	2.1	5.3
	Professional	2.3	2.9	3.0	2.9	3.0	3.1

**Figure 2:**
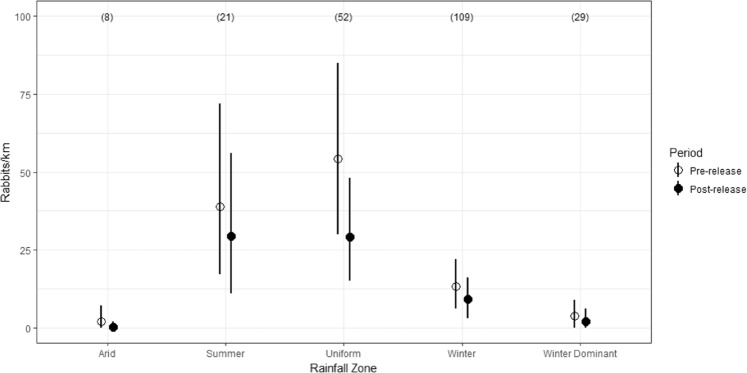
Mean rabbit abundance (rabbits/km) for pre- and post-release sampling times for each rainfall zone. Lines indicate the 95% credible intervals. Number of sites are given in parentheses.

Mean rabbit abundance (rabbits/km) was highest on sites in the uniform rainfall zone averaging 54 rabbits/km. Mean rabbit abundance was also high on sites in the summer rainfall zone averaging 39 rabbits/km (Fig. 2 and see Supplementary Information Table S1). Mean rabbit abundance was low in both the arid zone and winter dominant rainfall sites (Fig. 2). However, as there were only 8 sites in the arid rainfall zone, estimates of mean rabbit abundance could be atypical for this region, especially as 6 of the 8 sites were located in a single area on Exmouth gulf in Western Australia.

**Figure 3:**
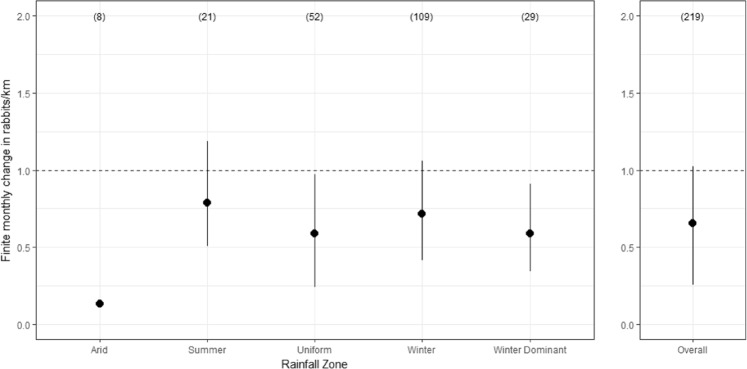
Mean finite monthly growth rate in rabbit abundance for each rainfall zone estimated from pre- and post-release spotlight monitoring data. Above the horizontal dashed line indicates population increase between pre- and post-release surveys, while below the dashed line indicates a decrease. Lines indicate the 95% credible intervals. Number of sites in parentheses.

**Figure 4:**
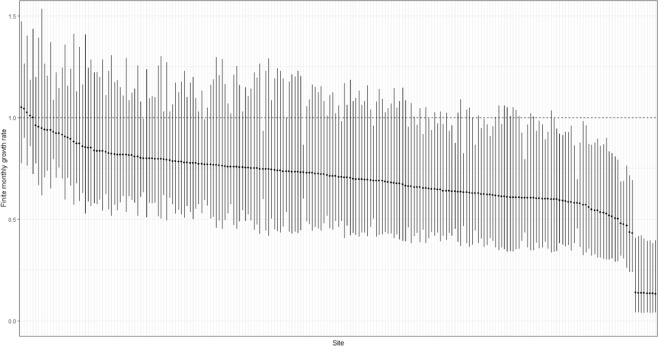
Finite monthly growth rate estimates for each of 219 citizen science monitored sites ordered from highest to lowest. Vertical lines indicate the 95% credible intervals. Above the horizontal dashed line indicates a population increase between pre- and post-release surveys, while below the dash line indicates a decrease.

Average rabbit abundance declined in all rainfall zones following the release of K5. However, average finite monthly growth rates were only significantly different from 1.0 (i.e. no change) for the arid, uniform and winter dominant zones (Fig. 3). Estimates of the average finite monthly growth rate in rabbit abundance following the release were lowest for the arid zone sites at 0.13, a monthly reduction of 87%. As previously mentioned, this estimate was dominated by 6 sites from a single area and hence, could be atypical. For the other rainfall zones, the lowest finite monthly growth rate was recorded for the uniform rainfall zone, averaging 0.59 (95 CI; 0.24–0.97), a mean monthly reduction of 41%. The highest finite monthly growth rate was recorded for sites in the summer rainfall zone, with an average growth rate of 0.79 (95% CI, 0.51, 1.19), an average monthly reduction of 21% (Fig. 3). The overall finite monthly growth rate in rabbit abundance was estimated as 0.66 (95%CI, 0.26, 1.03), averaging a monthly reduction of 34% (Fig. 3). Of the 219 citizen science-managed sites, spotlight data indicates that 204 (93%) exhibited a decrease in rabbit abundance following the release of K5, while 95 (44%) showed evidence of a significant decrease in the finite growth rate. Conversely, no sites registered a significant increase (Fig. 4).

### Professional data

**Figure 5:**
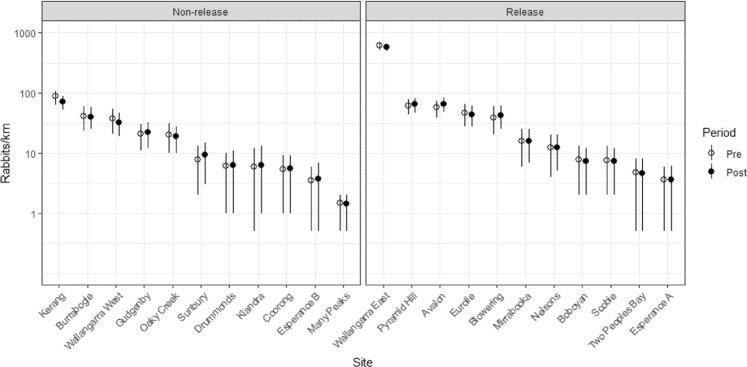
Rabbit abundance estimates (rabbits/km) for each of the K5 release and non-release (control) professionally-monitored sites for the pre- and post-release survey periods. Vertical lines indicate the 95% credible intervals. Rabbit abundance estimates are presented on the log_10_ scale.

**Figure 6:**
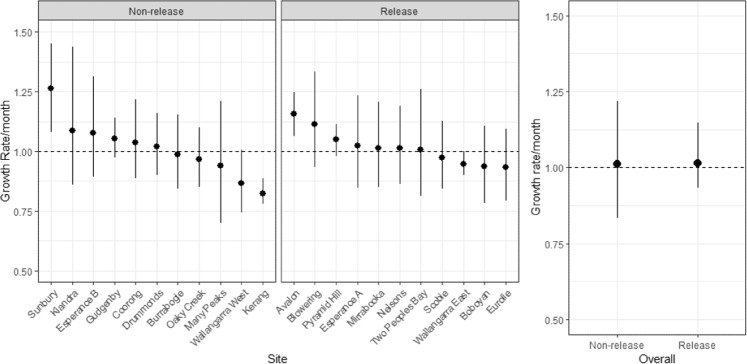
Finite monthly growth rate estimates for each of the K5 release and non-release (control) professionally monitored sites. Above the horizontal dashed line indicates a population increase between pre- and post-release surveys, while below the dash line indicates a decrease. Vertical lines indicate the 95% credible intervals.

For the 22 professionally monitored sites, rabbit abundances ranged from 612 rabbits/km to 1.5 rabbits/km (Fig. 5 and see Supplementary Information Tables S2 and S3). Four of the K5 release sites exhibited declines in rabbit abundances following the release relative to their pre-release abundances (Fig. 6). However, only one of these declines was significant based on the estimates of finite monthly growth rates having 95% credible intervals that excluded 1.0 (Fig. 6). Conversely, three K5 release sites showed evidence of increases between pre- and post-release survey periods with one of these being significant (Fig. 6). For the non-release sites, one site showed evidence of a significant decline between pre- and post-release survey periods with one site showing a significant increase (Fig. 6). Overall estimates of the finite monthly growth rates were approximately equal to 1.0 for both K5 release sites and non-release sites, indicating no substantial changes occurred between pre and post-release survey periods, on average (Fig. 6).

### Comparisons between citizen science and professionally monitored data

**Figure 7:**
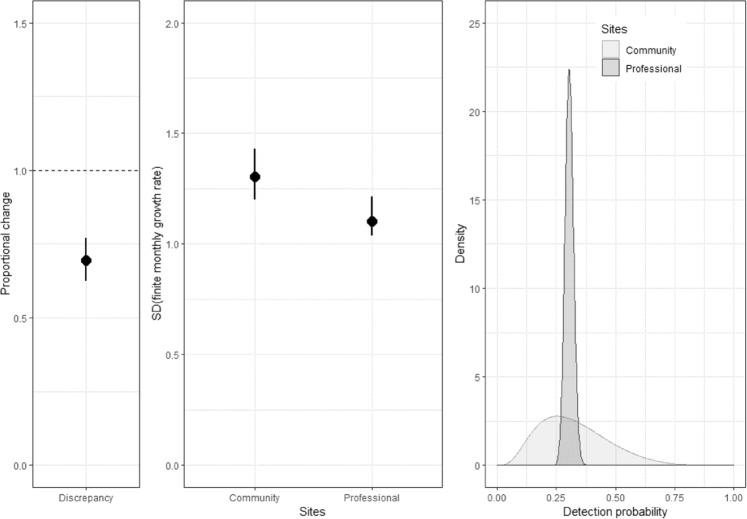
The estimate of the discrepancy in the average finite monthly growth rate between citizen science and professionally monitored sites (left panel), the estimated standard deviation (SD) of the average finite monthly growth rates (middle panel) and the distributions of detection probabilities (right panel).

The estimate of the average discrepancy in the finite monthly growth rate between citizen science and professionally monitored sites (*κ* – Eq. 4) had a ratio that was significantly less than 1.0 (0.68; 95% CI, 0.60–0.77). This indicates that the growth rate estimates on citizen science sites were 32% lower than on professional monitored sites, on average (Fig. 7).

In addition, the standard deviation of the average growth rate was 18% higher on citizen science sites compared with professional sites, indicating higher variation in the growth rate measured on citizen science sites compared to professional monitoring sites (Fig. 7). This higher variation also extended to estimate of the detection probability of rabbits where variation in the detection probability was over eight times higher on citizen science sites compared with professional sites (standard deviations of 0.71 vs 0.08) (Fig. 7).

## Discussion

Analysis of the citizen science monitoring data to estimate the initial impact of the K5 release from 219 sites across Australia suggests that the release of the K5 strain corresponded to an initial impact on rabbit populations amounting to an average 34% reduction in population abundance over one month. Rabbit abundance exhibited high variability both within and between rainfall zones and average monthly proportional changes indicated decreases in all zones, especially in the arid, uniform and winter-dominant rainfall zones. Over 90% of citizen science monitored sites recorded decreases in rabbit abundance following the release, with 32% of those recording significant reductions.

In contrast, similar monitoring conducted at 22 professionally monitored sites indicated that the release of the K5 strain had negligible impact on rabbit abundance with no K5 release sites recording significant decreases and similar changes in rabbit abundance on both release and non-release sites. Overall, the average decrease in rabbit populations on citizen science sites was 32% greater and 18% more variable than on professionally monitored sites.

Previous studies examining the utility of spotlight surveys of nocturnal animal populations by citizen scientist volunteers have shown that abundance estimates can have high bias compared with spotlight surveys carried out by experienced observers, with bias increasing with age and lack of previous experience^21^. Spotlight surveys on citizen science sites in this study had estimated detection probabilities that were unbiased compared to detection probabilities on professional sites with a mean of approximately 0.45. However, the variation in detection probability was over 6 times that on professional sites. In addition, detection probability estimates on citizen science sites differed little between pre- and post-release survey periods. Given the lack of bias in spotlight detection probabilities compared to professional monitored sites or between survey periods, it seems that systematic variation in spotlight surveys conducted on citizen science sites was insufficient to explain the large discrepancy in the estimated impact of K5 between citizen science and professional sites.

A reason for the discrepancy in the estimated impact of K5 between citizen science and professional sites could be that professional sites were unrepresentative of the rabbit populations and environmental conditions sampled by citizen science sites. However, the locations of the professionally monitored sites covered a similar spatial scale to the citizen science sites so it is difficult to argue that the results on professional sites are unrepresentative of the biogeographic factors that could have affected citizen science sites. Estimates of initial rabbit abundance on professional sites were also within a similar range to that estimated on citizen science sites, with the exception that more citizen science sites had low rabbit abundances (<2.7 rabbits/km) compared to professional sites. The citizen science sites had a similar proportion of high abundance sites compared with professional sites, so an excess of high abundance sites cannot explain the far greater estimated impact of the K5 release on citizen science sites. An alternative explanation is there were an insufficient number of professional sites to detect a change, particularly in the wake of the arrival of RHDV2 in Australia.

RHDV2 spread rapidly across Australia after its arrival (see^22^) and was detected at every intensive site prior to the release of K5 (unpublished data), with the observed average reduction in population equilibrium abundances of 60% attributed to its arrival (Ramsey *et al*., in review). Despite the arrival of RHDV2, the decision was made to proceed with the release of K5 to maintain the high level of public momentum in rabbit management that was associated with the release. The release and monitoring of K5 went ahead at intensive sites regardless of the impact of RHDV2 and the remaining population size. However, at the citizen science sites, those sites without rabbits withdrew from the release. This could mean that those sites that participated had a rabbit population that had not been, or had not sufficiently been, affected by RHDV2. We do not have pre-release serology for the citizen science sites, so it is impossible to determine how many of those sites that went ahead with the release had populations that had not been exposed to RHDV2. Given that we still do not fully understand the interactions of RHDV2 and K5, it is difficult to speculate on the true impact of RHDV2 on the K5 release. Further research to tease out the interactions between RHDV2 and K5 (and other caliciviruses present in Australia) is ongoing.

One key issue with the citizen science data was that complete data was only obtained for 219 of the possible 738 citizen science sites that remained in the K5 release program (i.e. 30%). Hence, there was a high rate of non-completion of the monitoring protocol that was expected to be undertaken in exchange for receiving a complementary vial of the K5 virus. An extensive community engagement program was conducted leading up to the release with citizen science groups given training material outlining methods for spotlight surveys and methods for inoculating the rabbit population with the K5 virus. Thus, it seems unlikely that the monitoring program was not completed because of a lack of access to training or instructional material.

Reasons for the high rate of non-completion may include de-motivating factors such as time constraints on the participant^23,24^ or expectation bias. Expectation bias is a well-known phenomenon where assessors observe or interpret their observations in a way that supports their prior expectations^25,26^. These cognitive biases may be conscious (i.e. fraud or fabrication) or unconscious^26,27^. To eliminate or control expectation bias requires that the assessors be blinded to information or conditions that could be expected to bias observations (i.e. be blind to the treatment condition applied or the hypotheses being tested)^27^. For the citizen science assessment of the K5 release, blinding could have been undertaken by distributing placebo vials of K5 (i.e. vials that contained an inert substance instead of the virus) to a random sample of the citizen science sites without their knowledge. However, this was considered undesirable due to the perception that trust in the agencies undertaking community engagement around rabbit issues would be undermined if knowledge about the blinding came to light. This perception was unfortunate as it has resulted in an inability to draw valid conclusions about the wider initial impact of the K5 release from this large-scale, citizen science dataset.

The rise of citizen science has enabled members of the public and the scientific community to work together to help understand environmental issues and citizen science data are now increasingly being used to monitor and model the distribution and abundance of wildlife populations (https://citizenscience.org.au/). There is no doubt that citizen science programs have made considerable contributions to ecological research programs in fields such as landscape ecology and climate change, urban and agricultural ecology, population dynamics and species distributions^1,4^. However, while acknowledging the contributions that citizen science has made to ecological research, scientists still need to be aware of the potential limitations of citizen science data. Citizen science has undoubtedly resulted in access to a huge repository of spatially and temporally extensive data, yet there is still a need for critical evaluation of such data sources before they can be seriously considered for management purposes.

While citizen science programs can make useful contributions to scientific research, the reliability of citizen science datasets must be evaluated to ascertain the size of likely biases that could influence the conclusions drawn from these data. Unfortunately, the release of K5 was confounded by the presence of the exotic virus RHDV2 and its rapid spread through Australia’s wild rabbit population in the 6 months prior to the K5 release^22^. Similarly, the lack of blinding (due to the decision not to use placebo vials), makes it difficult to tease apart any unconscious bias that might be present in the citizen science-submitted data. To improve the use of citizen scientists in research having experimental aspects (i.e. hypothesis testing/treatment evaluation), we strongly advocate the use of blind observations to mitigate the effects of any unconscious biases that could influence the results.

Despite being unable to confidently tease out the discrepancies in the results between the citizen science- and professionally-run sites and the true impact of K5, the program was considered a success from a community engagement perspective. Land managers from across the country participated in the release and submitted data and continue to provide disease data through the RabbitScan (http://www.feralscan.org.au/rabbitscan) resource. The ability to engage the community in a biological control program ensured that both virus distribution and data collection occurred over a vast area and in a variety of habitats; an outcome that would not have been possible without citizen scientist involvement. Further work on understanding the drivers behind why people participated and how they applied the best-practice knowledge in their ongoing rabbit management may yet reveal insights into how best to encourage and utilize citizen science in pest animal management on such large scales.

## Supplementary information

Supplementary Info

## Data Availability

The datasets generated during and/or analysed during the current study are available from the corresponding author on reasonable request.
