# Supplementary material for: The impact of RHDV-K5 on rabbit populations in Australia: an evaluation of citizen science surveys to monitor rabbit abundance
Source: Sci Rep. 2019 Oct 23;9:15229. doi: 10.1038/s41598-019-51847-w (PMC6811621; doi:10.1038/s41598-019-51847-w)
Supplement: Supplementary file 1 — Supplementary Info [file 41598_2019_51847_MOESM1_ESM.pdf]

The impact of RHDV1-K5 on rabbit populations in Australia: an evaluation of citizen science surveys to monitor rabbit abundance

Tarnya E Cox, David SL Ramsey, Emma Sawyers, Susan Campbell, John Matthews, and Peter Elsworth

Supplementary Information

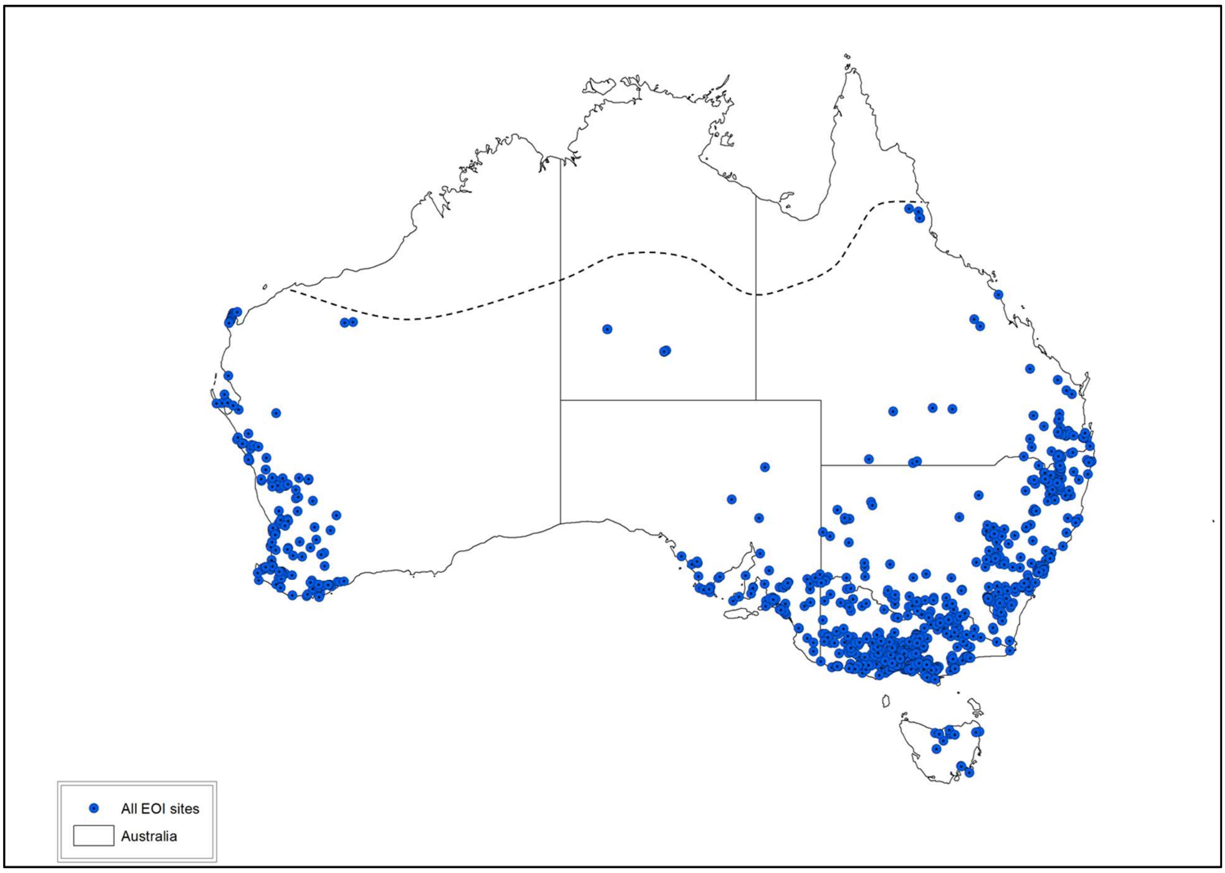

Supplementary Figure S1: The distribution of EOI sites received for the citizen science release of K5 (n=1066). Dashed line indicates northern limit of rabbit distribution. Map created in ArcGIS Desktop, Release 10.3.1.

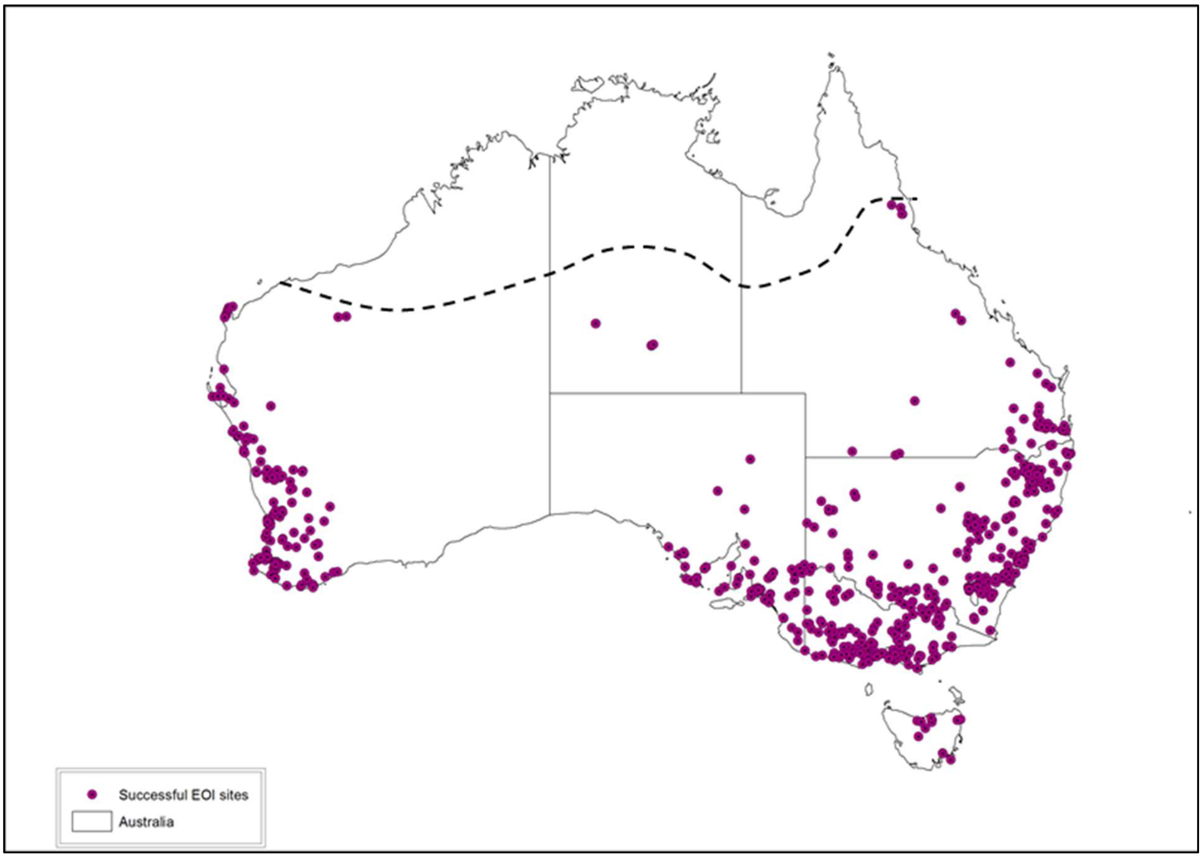

Supplementary Figure S2: The distribution of the successful EOI sites in the citizen science release of K5 (n=738). Dashed line indicates northern limit of rabbit distribution. Map created in ArcGIS Desktop, Release 10.3.1.

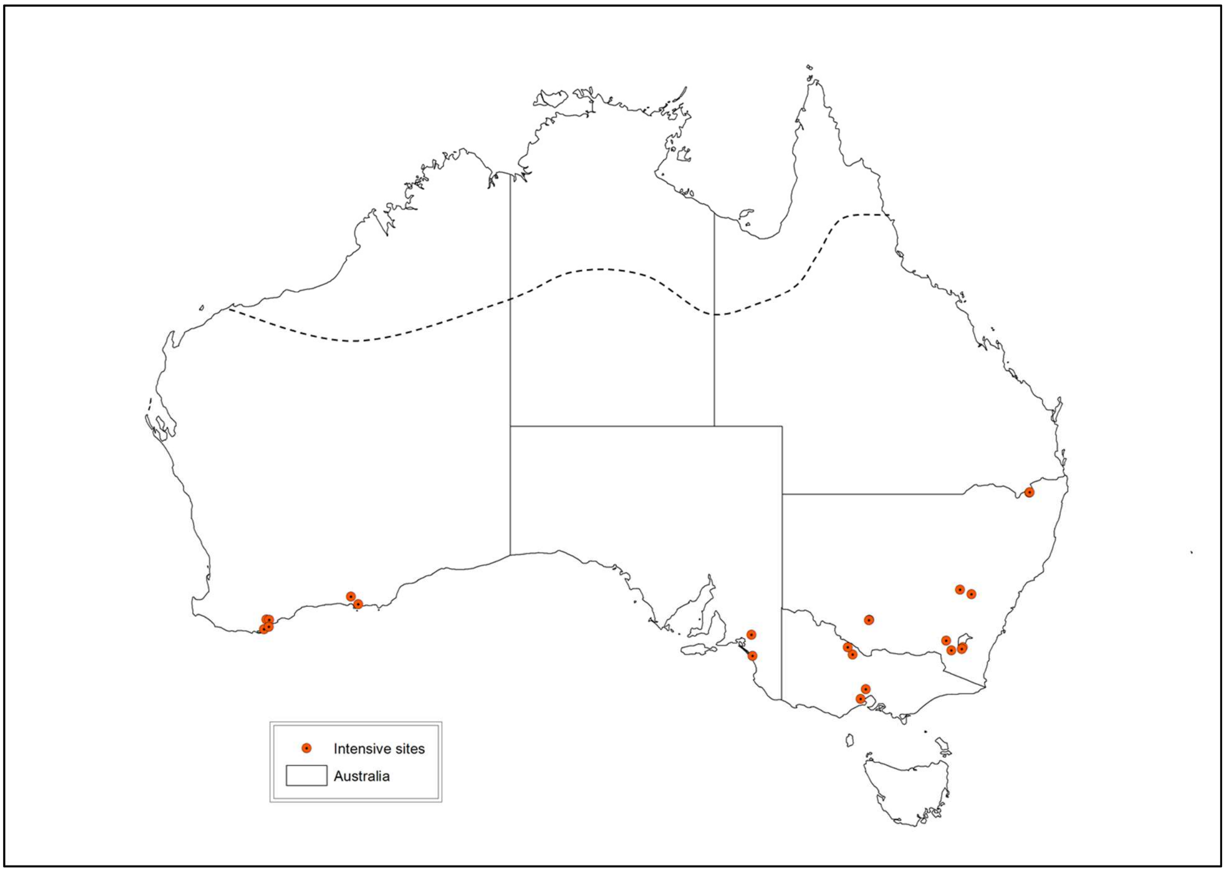

Supplementary Figure S3: Location of the 11 paired (one control and one release) State-run intensively monitored sites. Sites were sampled quarterly in the middle of each season for rabbit abundance and serological status. Dashed line indicates northern limit of rabbit distribution. Map created in ArcGIS Desktop, Release 10.3.1.

Supplementary Table S1: National and State Criteria used to select successful EOIs for the citizen science release of K5. All States/Territories had to follow the national criteria for site selection. The State Criteria is a list of additional criteria used by some states/territories to select their sites.

**National Criteria**	**State Criteria**
Proximity to national sites (50km buffer)	Maintaining community goodwill and participation regarding rabbit management
Areas of rabbit proneness	Encouraging adoption control programs, ownership for positive outcomes
Groups rather than individuals	Improved knowledge and understanding of rabbit management
RHDV K5 is a restricted chemical product – access to an authorised officer for all states and territories (except Victoria)	Ensure maximum distribution, priority to higher rabbit populations and assets of greater risk

Supplementary Table S2: Estimates of mean rabbit abundance (rabbits/km) for the pre-release and post-release sample times for each rainfall zone for the citizen science-managed sites. *n* = number of sites. 2.5% and 97.5% indicate the 95% credible intervals for the abundance estimates.

Zone	*n*	Pre-release	2.5%	97.5%	Post-release	2.5%	97.5%
Arid	8	2.2	0	7	0.3	0	2
Summer	21	39.2	17	74	29.7	12	57
Uniform	52	54.3	31	83	29.0	15	47
Winter	109	13.2	6	22	9.1	3	16
Winter-dominant	29	3.7	0	9	2.0	0	6

Supplementary Table S3: Estimates of mean rabbit abundance (rabbits/km) for the pre-release and post-release sample times for the professional monitored sites. 2.5% and 97.5% indicate the 95% credible intervals for the abundance estimates.

Site	State	Pre-release	2.5%	97.5%	Post-release	2.5%	97.5%
**Non-release** sites							
Gudgenby	ACT	20.2	11	30	21.6	12	32
Burrabogie	NSW	39.9	24	59	39.1	25	57
Kiandra	NSW	4.9	0	12	5.2	1	13
Oaky Creek	NSW	19.1	10	31	18.3	10	28
Wallangarra West	QLD	36.4	21	54	31.3	19	46
Coorong	SA	4.3	1	9	4.5	1	9
Kerang	VIC	87.0	64	108	71.1	53	89
Sunbury	VIC	6.8	2	13	8.4	3	15
Drummonds	WA	5.2	1	10	5.4	1	11
Esperance B	WA	2.5	0	6	2.7	0	7
Many Peaks	WA	0.5	0	2	0.4	0	2
**Release sites**							
Boboyan	ACT	6.9	2	13	6.4	2	12
Blowering	NSW	37.6	20	59	41.5	25	61
Eurolie	NSW	46.3	28	66	43.1	28	62
Mirrabooka	NSW	14.9	6	25	14.9	7	25
Wallangarra East	QLD	611.0	517	691	577.7	501	648
Scobie	SA	6.7	2	13	6.4	2	12
Avalon	VIC	56.0	39	74	64.9	48	83
Pyramid Hill	VIC	60.5	44	78	63.7	47	81
Esperance A	WA	2.6	0	6	2.6	0	6
Nelsons	WA	11.3	4	20	11.3	5	20
Two Peoples Bay	WA	3.7	0	8	3.7	0	8
